# Serum interleukin‐10 as a valuable biomarker for early diagnosis and therapeutic monitoring in intravascular large B‐cell lymphoma

**DOI:** 10.1002/ctm2.131

**Published:** 2020-07-07

**Authors:** Yan Zhang, Liang Wang, Jian Sun, Wei Wang, Chong Wei, Daobin Zhou, Wei Zhang

**Affiliations:** ^1^ Department of Hematology, Peking Union Medical College Hospital Chinese Academy of Medical Sciences & Peking Union Medical College Beijing China; ^2^ Department of Hematology, Beijing Tongren Hospital Capital Medical University Beijing China; ^3^ Beijing Advanced Innovation Center for Big Data‐Based Precision Medicine Beijing Tongren Hospital Beihang University & Capital Medical University Beijing China; ^4^ Department of Pathology, Peking Union Medical College Hospital Chinese Academy of Medical Sciences & Peking Union Medical College Beijing China

Dear Editor,

Intravascular large B‐cell lymphoma (IVLBCL) is a relatively rare and distinct subtype of diffuse large B cell lymphoma (DLBCL) and difficult to diagnose due to lacking lymphadenopathy or specific tumor markers.^1^ Detection of diverse cytokines in serum has been found to play a significant role in understanding the relationship between the immune system and the host in various types of hematological tumors. By mediating an immunosuppressive tumor microenvironment, IL‐10 could facilitate tumor growth and immune evasion.^2^ IL‐10 level in cerebrospinal fluid has been demonstrated to represent a potential diagnostic biomarker for central nervous system (CNS) lymphoma,^3^ and pretreatment serum IL‐10 could predict the CNS relapse risk in DLBCL.^4^ Due to the extremely high risk of CNS involvement in patients with IVLBCL both at diagnosis and relapse,^5^ we supposed that IL‐10 may also play a crucial role in IVLBCL development. Herein, we uncovered the role of serum IL‐10 as a valuable biomarker for early diagnosis and therapeutic monitoring in IVLBCL.

Thirty‐five consecutive patients with IVLBCL between 2010 and 2019 were analyzed. Baseline serum IL‐10 level was measured using an electrochemiluminescence immunoassay analyzer (Siemens Immulite 1000 and its corresponding IL‐10 detection kit) in those 35 patients with IVLBCL and 203 consecutive patients admitted in the department of Hematology, Rheumatology and Infectious disease in PUMC hospital from April to June 2019. The Institutional Review Board of PUMC hospital approved this study.

As is shown Table S1, 33 patients (94.3%) were categorized in the high‐risk or intermediate‐high‐risk group, and nine (25.7%) had hemophagocytic lymphohistiocytosis. Most patients had more than one extranodal lesions with a median of 3 (range 1‐6), and bone marrow, lung, spleen, and skin in order were the most involved organs. A total of 87.0% patients were non‐GCB subtype, and 66.7% (12/18) patients were tested positive for CD5. Eight patients were evaluated for both expressions of c‐Myc and Bcl‐2, three of whom were confirmed as having double‐expressers lymphoma. Moreover, IL‐10 was positive in all 17 patients who were tested (Figure S1). In the control group of 203 patients, 53 were hospitalized for fever of unknown reason (FUO), 74 were diagnosed as various connective tissue disease (CTD), and 76 were confirmed to be non‐lymphoma hematologic disease. As shown in Figure 1, the serum levels of IL‐10 in IVLBCL were significantly higher than those in control groups (*P* < .0001), with a median level of 490 pg/mL (range, 5‐1000) versus 5 pg/mL (range, 5‐75.3). Moreover, IL‐10 level was significantly higher in IVLBCL than in DLBCL, not otherwise specified (DLBCL‐NOS) (*P* = .0005). The ability of serum IL‐10 to identify IVLBCL from all suspected disease was assessed by ROC curves, and it had a good performance in identifying IVLBCL with area under the curve of 0.915 (95% confidence interval [CI], 0.811‐1). When the cutoff level of serum IL‐10 was defined as 95.65 pg/mL, the diagnostic sensitivity and specificity for IVLBCL were 80% and 100%, respectively. In order to determine the role of serum IL‐10 in therapeutic monitoring, we compared the pre‐ and posttreatment levels of IL‐10 in those patients with IVLBCL. All patients had decreased IL‐10 levels after treatment, but only those who got complete remission (CR) had their IL‐10 level decreased to lower than 30 pg/mL.

**FIGURE 1:**
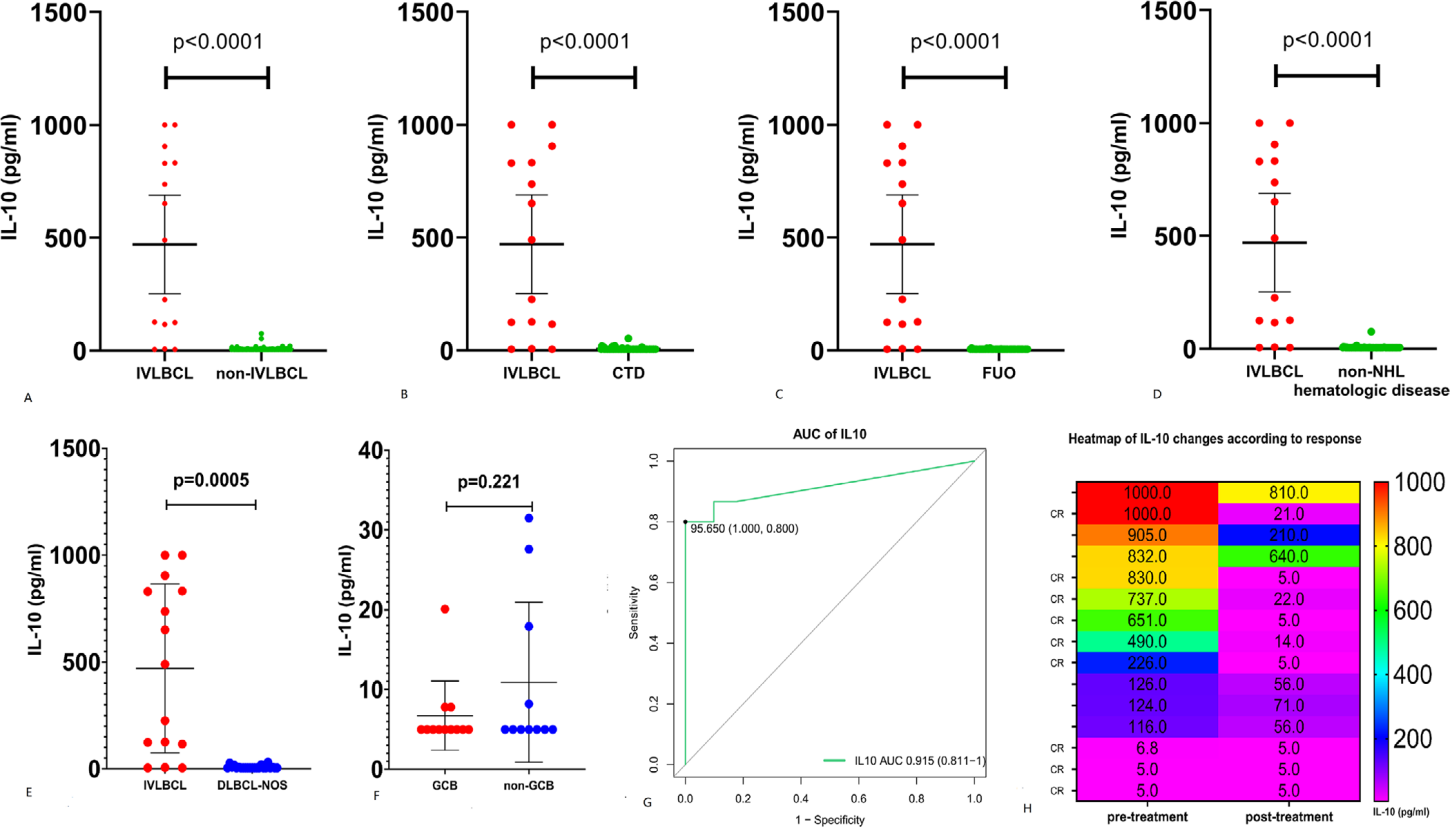
Serum level of IL‐10 in patients with IVLBCL and non‐IVLBCL disease. A, Serum levels of IL‐10 in 15 patients with IVLBCL were significantly higher than those in 203 patients with non‐IVLBCL disease (*P* < .0001). B, Serum levels of IL‐10 in 15 patients with IVLBCL were significantly higher than those in 74 patients with CTD (*P* < .0001). C, Serum levels of IL‐10 in 15 patients with IVLBCL were significantly higher than those in 53 patients with FUO (*P* < .0001). D, Serum levels of IL‐10 in 15 patients with IVLBCL were significantly higher than those in 76 patients with non‐lymphoma hematologic disease (*P* < .0001). E, Serum levels of IL‐10 in 15 patients with IVLBCL were significantly higher than those in 23 patients with DLBCL‐NOS (*P* = .0005). F, No significant difference was found between GCB and non‐GCB subtype of DLBCL‐NOS. G, ROC curve indicated good performance of serum IL‐10 in diagnosing IVLBCL with an area under the curve of 0.915 (95% CI, 0.811‐1). H, Dynamic changes of serum IL‐10 level at the time of pre‐ and posttreatment

CHOP regimen with and without rituximab was administrated in 23 and four patients, respectively, and additional intravenous methotrexate (MTX) was administered to 63.0% patients (17/27) for CNS prophylaxis or treatment of CNS involvement. Treatment responses were evaluated in all patients who received chemotherapy, CR was achieved in 19 (70.4%) of 27 patients, and five (18.5%) patients achieved partial remission (PR). The CR rate was significantly higher in those received intravenous MTX (88.2% vs 40.0% in those who did not receive intravenous MTX, *P* = .025). As of 1 April 2020, the median follow‐up time was 42.4 months (5.7‐120.0). All 35 patients had detailed overall survival (OS) data, with 3‐year OS rate being 59.5% (95% CI, 40.0‐74.6%). No secondary CNS involvement occurred in patients treated with HD‐MTX. As is shown in Figure 2, four factors were found to be significantly correlated with OS in patients with IVLBCL, namely, age (*P* = .049), IPI (*P* = .036), intravenous high‐dose MTX (*P* = .002), and treatment responses (*P* < .001). As is shown in Table S2, multivariate COX regression model found that age (>60), no administration of intravenous HD‐MTX, and no achievement of CR were found to be independent poor prognostic factors.

**FIGURE 2:**
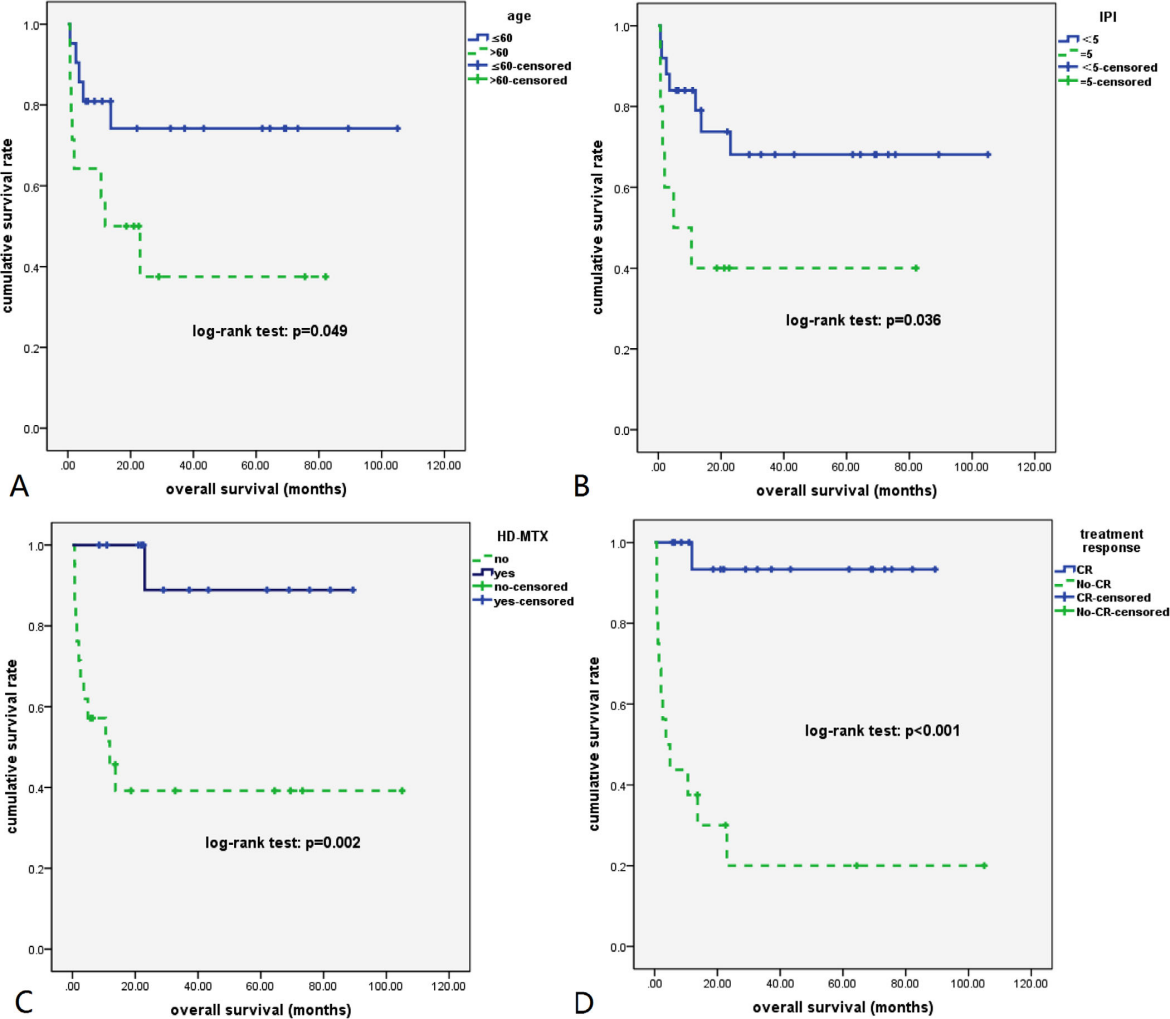
Survival outcomes for all 35 patients with IVLBCL. A, Patients older than 60 years old had significantly inferior overall survival (*P* = .049). B, Patients with IPI score of 5 fared worse than those with IPI < 5 (*P* = .036). C, Patients that did not receive HD‐MTX had significantly poor survival outcomes (*P* = .002). D, Patients that did not achieve complete remission had significantly inferior overall survival (*P* < .001)

Serum biochemical markers have been widely used to diagnose or predict prognosis in various tumors, such as the role of lactate dehydrogenase in lymphoma.^6^, ^7^ One of the highlights of our study was to uncover the value of serum IL‐10 in early diagnosis and therapeutic monitoring for IVLBCL. Compared with FUO, CTD, and other hematologic disease, serum IL‐10 was significantly higher in IVLBCL, and it had an excellent performance in diagnosing IVLBCL. Moreover, the dynamic changes of serum level of IL‐10 correlated with treatment responses, indicating it might be regarded as a biomarker of minimal residual disease for IVLBCL. CD5 was positive in 66.7% patients with IVLBCL, much higher than DLBCL‐NOS, which only accounts for about 10% of all DLBCLs.^8^ It is demonstrated that CD5‐positive DLBCL usually presents with advanced stages, frequent extranodal involvement, and had a very poor prognosis.^8^

CNS relapse risk in patients with IVLBCL was not reduced significantly even in the era of rituximab, which was reported to be 22% at 3 years.^9^ The PRIMEUR‐IVL study evaluated the efficacy of R‐CHOP combined with HD‐MTX in 37 patients of IVLBCL,^5^ concluding a CR rate of 84% and 2‐year OS rate of 92%, and also the risk of CNS relapses was drastically reduced to 3% at 2 years. Thus, both the results of our study and PRIMEUR‐IVL study underscored the importance of HD‐MTX in the treatment of IVLBCL.

In conclusion, this study described clinicopathological profiles of the largest IVLBCL group at a single institution in China. We defined the role of serum IL‐10 as a valuable biomarker for early diagnosis and therapeutic monitoring, and confirmed the benefits of HD‐MTX in both preventing CNS lesions and improving prognosis in patients with IVLBCL.

## ETHICS APPROVAL AND CONSENT TO PARTICIPATE

This study was approved by the Institutional Review Board (IRB) of PUMCH.

## AVAILABILITY OF DATA AND MATERIALS

The datasets supporting the conclusions of this article are included within the article. Yan Zhang and Liang Wang had full access to all the raw data in the study (available upon data specific request). All of the methods including the software programs or reagents used in this study are on the market, which are accessible by other researchers.

## CONFLICT OF INTEREST

The authors declare no conflict of interest.

### AUTHOR CONTRIBUTIONS

DBZ and WZ designed the study. YZ and LW collected and analyzed the data and wrote the manuscript. JS performed pathological research. WW and CW detected IL‐10 levels and collected the data. All authors read and approved the final manuscript.

## Supporting information

FigureS1

FigureS1.docx

TableS1.docx

TableS2.docx
