# Supplementary figures and images for: Serum interleukin‐10 as a valuable biomarker for early diagnosis and therapeutic monitoring in intravascular large B‐cell lymphoma
Source: Clin Transl Med. 2020 Jul 7;10(3):e131. doi: 10.1002/ctm2.131 (PMC7418806; doi:10.1002/ctm2.131)

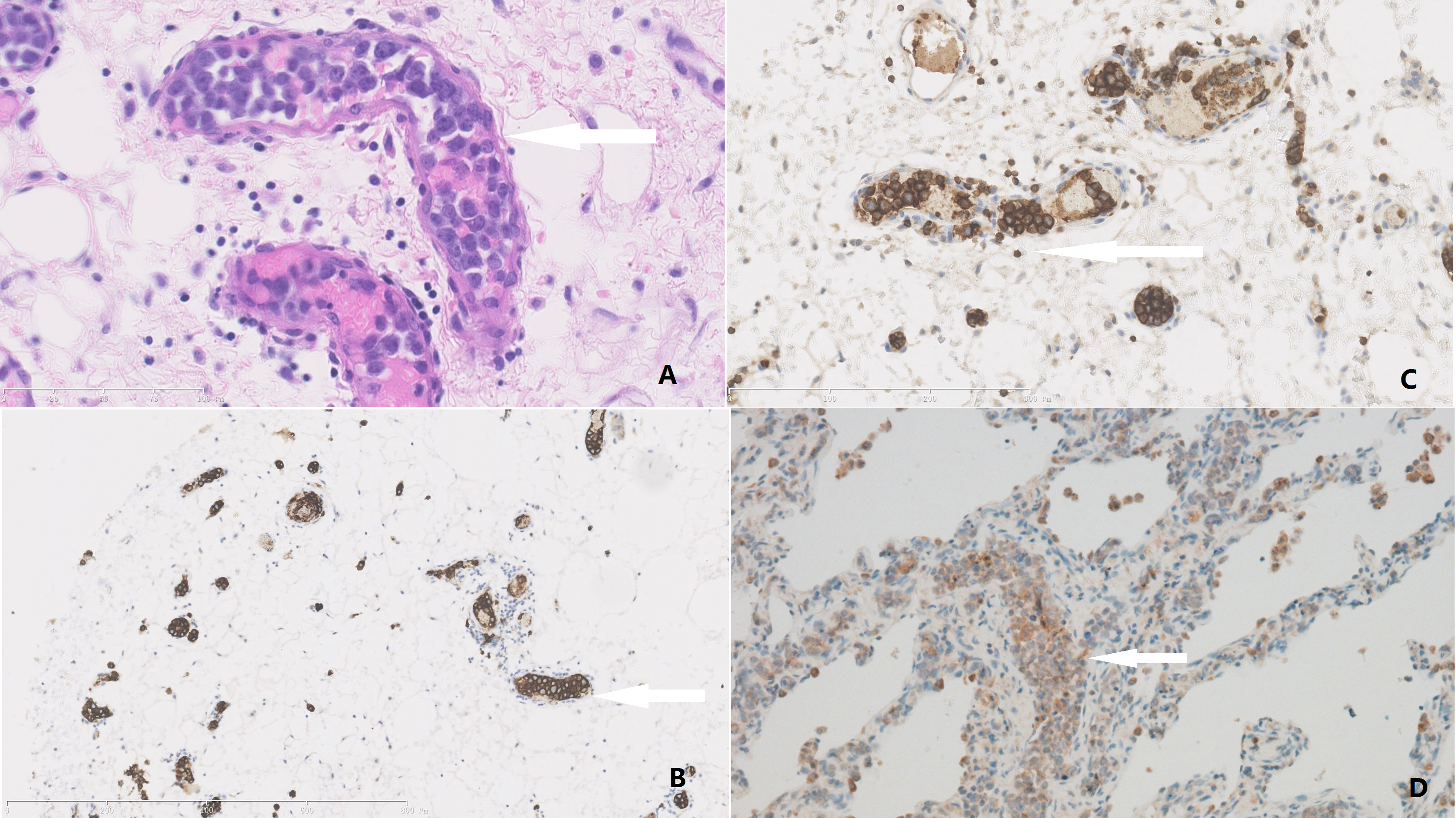

Supplement: Supplementary file 1 — FigureS1 [file CTM2-10-e131-s001.tif]
