# Supplementary material for: Serum interleukin‐10 as a valuable biomarker for early diagnosis and therapeutic monitoring in intravascular large B‐cell lymphoma
Source: Clin Transl Med. 2020 Jul 7;10(3):e131. doi: 10.1002/ctm2.131 (PMC7418806; doi:10.1002/ctm2.131)
Supplement: Supplementary file 2 — FigureS1.docx [file CTM2-10-e131-s002.docx]

**Supplemental Figure 1. Pathologic features of IVLBCL.**

A. HE staining showed large lymphoid cells within vessel lumina, and these lymphoma cells were large, with scant cytoplasm, vesicular nuclei and one or more nucleoli.

B. IHC staining for CD20 revealed universally positive.

C. IHC staining for CD5 revealed intensive positivity.

D. IHC staining for IL-10 revealed diffuse cytoplasmic positivity.
