# Supplementary material for: Serum interleukin‐10 as a valuable biomarker for early diagnosis and therapeutic monitoring in intravascular large B‐cell lymphoma
Source: Clin Transl Med. 2020 Jul 7;10(3):e131. doi: 10.1002/ctm2.131 (PMC7418806; doi:10.1002/ctm2.131)
Supplement: Supplementary file 3 — TableS1.docx [file CTM2-10-e131-s003.docx]

Table 1. Clinical characteristics and laboratory findings in patients with IVLBCL (N=35).

|  | No. | % |
| --- | --- | --- |
| Median Age at diagnosis |  |  |
| Gender，male | 18 | 51.4 |
| ECOG-PS＞1 | 23 | 65.7 |
| Extranodal involvement ＞1 | 30 | 85.7 |
| IPI score |  |  |
| 3 | 10 | 28.6 |
| 4 | 13 | 37.1 |
| 5 | 10 | 28.6 |
| B symptoms | 31 | 88.6 |
| Organ involvement |  |  |
| Bone marrow | 15 | 42.9 |
| Lung | 14 | 40.0 |
| Spleen | 14 | 40.0 |
| Skin | 13 | 37.1 |
| Liver | 8 | 22.9 |
| CNS | 7 | 20.0 |
| Bone | 5 | 14.3 |
| Adrenal gland | 4 | 11.4 |
| Kidney | 4 | 11.4 |
| HLH | 9 | 25.7 |
| Anemia | 24 | 68.6 |
| Thrombocytopenia | 20 | 57.1 |
| Leukocytopenia(<4.0×10^9^/L) | 15 | 42.9 |
| Elevated LDH | 34 | 97.1 |

Abbreviations: IVLBCL, intravascular large B cell lymphoma; ECOG-PS, eastern cooperative oncology group, performance status; IPI, international prognostic index; HLH, hemophagocytic lymphohistiocytosis; LDH, lactate dehydrogenase.
