# Supplementary material for: Serum interleukin‐10 as a valuable biomarker for early diagnosis and therapeutic monitoring in intravascular large B‐cell lymphoma
Source: Clin Transl Med. 2020 Jul 7;10(3):e131. doi: 10.1002/ctm2.131 (PMC7418806; doi:10.1002/ctm2.131)
Supplement: Supplementary file 4 — TableS2.docx [file CTM2-10-e131-s004.docx]

Table 2. Univariate and multivariate COX regression analysis.

| Parameters | Univariate COX analysis | | Multivariate COX analysis | |
| --- | --- | --- | --- | --- |
|  | HR (95% CI) | P value | HR (95% CI) | P value |
| Age (>60) | 2.920  （0.952-8.953） | 0.061 | 8.843  (1.565-49.974) | 0.014 |
| Gender (male) | 1.480  （0.492-4.450） | 0.485 |  |  |
| IPI (=5) | 3.075  （1.016-9.304） | 0.047 | 0.827  (0.170-4.025) | 0.814 |
| ECOG-PS (>1) | 0.857  (0.280-2.626) | 0.787 |  |  |
| B symptoms (presence) | 1.706  (0.221-13.149) | 0.608 |  |  |
| Extranodal sites (>2) | 1.584  (0.517-4.851) | 0.421 |  |  |
| Cytopenia (presence) | 0.631  (0.139-2.856) | 0.550 |  |  |
| HLH (presence) | 1.544  (0.474-5.030) | 0.471 |  |  |
| IV HD-MTX (no) | 18.681  (2.407-144.983) | 0.005 | 21.743  (2.127-222.259) | 0.009 |
| Responses (non-CR) | 23.906  (3.083-185.394) | 0.002 | 10.425  (1.150-94.521) | 0.037 |

Abbreviations：ECOG-PS, Eastern Cooperative Oncology Group-performance status; IPI, international prognostic index; HLH, hemophagocytic histiocytosis; IV, intravenous; HD-MTX, high-dose methotrexate; CR, complete response
